# Zimssen’s Cyclopædia, Vol. X

**Published:** 1875-10

**Authors:** 


### Books Reviewed.

*Cyclopædia of the Practice of Medicine.* Edited by Dr. H. von Ziemssen. Vol. X. *Diseases of the Female Sexual Organs.* By Prof. Carl Schrœder. American Translation. Edited by Albert Buck, M. D. New York: Wm. Wood & Co. 1875.

In compliance with the plan pursued by the publishers of the German edition of this work, Messrs. Wood & Co. have issued volume ten prior to the publication of volume four. Devoted to a subject which in the last few years has been recognized as a large and important field of special practice, it will attract as much attention as any volume of the series.

The entire volume is from the pen of Prof. Schrœder, who is already well known to American readers through his excellent manual of midwifery.

The introductory pages are devoted to a description of the proper methods of making vaginal examinations. In these he describes some of the more common and valuable specula, and expresses his preference in ordinary examinations for the tubular variety. In operations he employs the one invented by Simon, which is something after the general plan of Sim's.

He passes from the subject of examinations to that of diseases and malformations of the uterus. The malformations of that organ are briefly discussed and are illustrated by well-made wood-cuts of the more important varieties. The subjects of atresia, hypertrophys, atrophy and inflammations follow next in order. The consideration of the varieties of erosions and ulcers will be found to be particularly interesting, as it is this class of disorders which is more generally met with in every-day practice. The various forms of displacements and their treatment follow next in order. The mechanical treatment is recommended, and various pessaries and supporters figured which may be employed. In prolapse the author does not speak with much confidence of the operation for narrowing the vagina, which has at various times been recommended. The success of American surgeons in reducing old inversions of the uterus is recognized, and brief mention made of the success of Prof. White, of this city, and the instrument employed by him. Uterine fibroids and cancer are well described, several pages being devoted to their consideration ; following these subjects are a few pages only, on the important subject of menstruation. A little over nine pages are allotted to the diseases of the fallopain tubes, and then follows a consideration of the diseases of the ovary. Malformations, inflammations and displacements of that organ are first considered, after which the subjects of cysts and cystomata are taken up.

Schrœder recognizes two distinct varieties of cystic formation, dropsy of the Graafian follicle and the cystic tumor or cystoma. The dropsy of the Graafian follicle he regards as representing the retention cyst found in other glandular localities, while the cystoma is a new formation with a cystic formation arising from the follicles of the ovary. In the history of the operation, due credit is given to Dr Ephraim McDowell, of Kentucky, as the first to perform this operation in a rational manner and for a definite purpose. The author favors the extra-peritoneal treatment of the pedicle by means of the clamp. He says that the method of ovariotomy by enucleation, as first describod by Dr. Miner, of Buffalo, " deserves careful investigation," and gives a list of several surgeons who have used the method with success. In case the pedicle is short, he recommends that it be tied in several portions with catgut, the ligatures cut short and the stump returned to the abdominal cavity.

The balance of the work is taken up by a consideration of diseases of the uterine ligaments, adjacent portions of the peritoneum, and diseases of the vagina and vulva.

The work is well translated, and presents to English readers an admirable resume of the present status of Genæcology, perhaps more especially from a German standpoint, but in most respects, a slight modification will adapt it to American ideas.

